# Living Donor Liver Transplantation vs. Split Liver Transplantation Using Left Lateral Segment Grafts in Pediatric Recipients: An Analysis of the UNOS Database

**DOI:** 10.3389/ti.2022.10437

**Published:** 2022-03-22

**Authors:** Christina Dalzell, Paola A. Vargas, Kyle Soltys, Frank Dipaola, George Mazariegos, Jose Oberholzer, Nicolas Goldaracena

**Affiliations:** ^1^ School of Medicine, University of Virginia, Charlottesville, VA, United States; ^2^ Division of Transplant Surgery, Department of Surgery, University of Virginia Health System, Charlottesville, VA, United States; ^3^ Hillman Center for Pediatric Transplantation, UPMC Children’s Hospital of Pittsburgh and Department of Surgery, University of Pittsburgh School of Medicine, Pittsburgh, PA, United States; ^4^ Division of Pediatric Gastroenterology, Department of Pediatrics, University of Virginia, Charlottesville, VA, United States

**Keywords:** pediatric liver transplantation, outcomes, graft, waitlist, survival

## Abstract

Split and LDLT in pediatric patients have the potential to decrease wait times and waitlist mortality. Using UNOS-STAR data, we compared outcomes of pediatric patients undergoing LDLT and SLT using LLS grafts. The baseline characteristics and post-operative outcomes were compared between groups. Actuarial graft and patient survival were analyzed with Kaplan-Meier curves. Between 2010 and 2019, 911 pediatric LT were included in the analysis (LD graft group, *n* = 508, split graft group, *n* = 403). LD graft recipients spent more time on the waitlist vs. the split graft group (60 (22–138) days vs. 46 (16–108) days; *p* = 0.007). LD recipients had a lower rate of graft failure, found in 9.8% of patients compared with 14.6% in the split graft group (*p* = 0.02). HAT was the most common graft failure cause, with similar rates. Graft and patient survival at 1-, 3-, and 5-years was comparable between LDLT and SLT. In subgroup analyses, patients with biliary atresia, those ≤10 kg or ≤10 years old receiving an LD graft showed improved graft survival. In conclusion, LDLT is associated with a lower rate of graft failure in pediatric patients. The use of LLS regardless of the type of donor is a safe way to facilitate access to transplantation to pediatric patients with acceptable short and long-term outcomes.

## Graphical Abstract

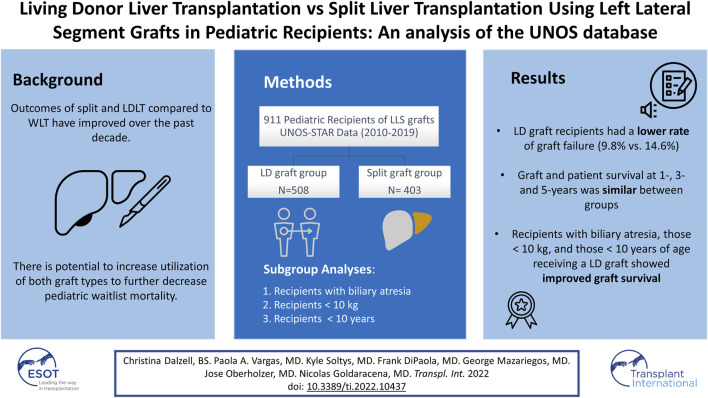

## Introduction

The use of split liver transplantation (SLT) and living donor liver transplantation (LDLT) have revolutionized the field of pediatric liver transplantation (LT)—with the potential to increase the availability of organs and decrease waitlist mortality (1). Pediatric LT creates a unique need for alternative graft types due to limited access to whole organs from pediatric deceased donors. Both techniques have created interest in better understanding the complications that arise and the factors that contribute to acceptable outcomes.

The number of pediatric LT performed in the US has remained stable over the past decade (2). Out of the 551 pediatric LT performed in 2019, LDLT and SLT accounted for 14.3% and 20.3% respectively, both increased from a decade prior (2). In 2019, 55% of pediatric candidates waited less than 1 year compared to 45.1% a decade prior (2). In this regard, over 90% of pediatric patients on the waitlist undergo LT, with a 5-years patient survival rate ranging from 81 to 93%, depending on the primary diagnosis (2, 3).

Deceased donor splitting into an LLS and an extended right graft, not only provides access to LT for pediatric recipients but also the possibility to utilize the remnant liver graft in a larger size recipient (pediatric or adult) (3, 4). The transplant community has recently supported the evaluation of a regional variance to support split liver transplantation within affiliated centers (5). The potential of a single organ to benefit two patients in need is countered by logistical challenges—including increased cold ischemia time, geographic barriers, surgical complexity, and manpower logistics (1). On the other hand, for LDLT, LLS is removed from a healthy donor (3, 4). Theoretical benefits of LDLT include faster access to transplantation, earlier timing of surgery before clinical decompensation, less cold ischemia time, expansion to patients who would otherwise not qualify for a deceased donor liver, and potential immunologic advantages in related donors (6–8).

Over the past decade, the outcomes of split and living donor vs. the whole LT in the pediatric population have improved (9). Some studies have shown a potential increase in graft failure and complications in SLT when compared to whole LT or LDLT, whereas other studies have shown no impact of graft type on graft or patient outcome (10, 11). Few studies have directly compared these techniques in a pediatric population, and studies comparing outcomes based on the type of grafts among these patients are even more scarce. This study aims to compare patient and graft outcomes in the pediatric population following LDLT and SLT using LLS grafts.

## Materials and Methods

### Study Design

Data for this retrospective study were obtained from the United Network for Organ Sharing (UNOS) Standard Transplant Analysis and Research file (STAR). The UNOS-STAR database does not include any patient or transplant center identifiers. The study population included all pediatric recipients (<18 years of age) who received a LLS graft from a deceased donor (SLT) or a living donor (LD) in the United States from January 1, 2010, to March 2019. Recipients ≥18 years, patients receiving a right lobe, right trisegment graft, full left lobes, whole liver grafts, donation after circulatory death, multi-organ transplants, re-transplantations, and those transplanted prior to 2010 were excluded from the analysis. The records of the remaining pediatric liver transplant recipients were then analyzed.

Three additional subgroup analyses were performed to assess outcomes with the two techniques in challenging pediatric populations. The first one compared the outcome of both techniques in pediatric patients with biliary atresia. While a second analysis evaluated recipients ≤10 kg, the third analysis assessed both techniques in those patients who were ≤10 years of age at the time of transplant. Demographic variables for donors, recipients, and postoperative outcomes were compared in each set of patients.

### Donor and Graft Data

The following donor characteristics were analyzed and compared between groups: age, gender, weight (kg), height (cm), body mass index (BMI), and cold ischemia time (CIT) (hours).

Since November 2007, the OPTN has identified organs with the potential to be split as those that met the following criteria: donor less than 40 years old on less than 1 vasopressor, transaminases no greater than 3 times the upper limit of normal, and BMI of 28 or less. However, although these are meant as guidelines, the final decision to split or not an organ is based on each transplant center’s criteria and expertise.

In the deceased donor group, some of the left lateral segment grafts resulted from a reduction of a full graft rather than a true split into two grafts. For descriptive purposes, we described these grafts also as splits although the extended right graft was not used for another recipient.

### Recipient Data

The following recipient data were analyzed and compared between groups: age, gender, weight (kg), height (cm), BMI, laboratory values at transplant such as international normalized ratio (INR), albumin level, serum creatinine, and total bilirubin level. Preoperative data such as ascites grade, history of portal vein thrombosis (PVT), previous upper abdominal surgery, indication for transplant, Pediatric End-Stage Liver Disease (PELD) score at transplant, patients transplanted under status 1, and the total number of days on the waitlist were also analyzed.

Status 1 variable was comprised of patients with Status 1A and 1B. Status 1A and 1B are the only medical priority exceptions to PELD scores in pediatric patients and account for less than 1% of liver transplant candidates at any given time. Status 1A patients are those with a diagnosis of acute liver failure with a life expectancy of less than 7-days. Status 1B includes patients with hepatoblastoma, certain metabolic disorders, and chronic liver disease with a MELD or PELD greater than 25.

### Postoperative Outcomes

Recipient surgical outcomes were analyzed by assessing the length of hospital stay (LOS), and the reported incidence of graft failure causes such as hepatic artery thrombosis (HAT), other vascular thrombosis, primary non-function (PNF), infection, PVT, biliary related graft failure, diffuse cholangiopathy, hepatitis *de novo*, recurrent disease and hepatic outflow obstruction. Re-transplantation rates and mortality were also analyzed by actuarial graft and patient survival.

### Statistical Analysis

Baseline donor and recipient demographics, as well as clinical characteristics, were presented as median (interquartile range) for continuous variables, and counts (percent) for categorical variables, unless stated otherwise according to the distribution of the data. For categorical variables, the *Chi-square* test and *Fisher’s Exact Test* were used for comparison between groups accordingly. *Independent t-test* and *Mann-Whitney U test* were used for comparisons of continuous variables as appropriate. Kaplan-Meier method was used to analyze survival between study groups and were compared using the log-rank test. The outcome for graft and patient survival were calculated by using the variables “pstatus” and “gstatus”-Boolean most recent patient status (based on composite death date)- respectively. These variables reflect the death date reported for the patient as deceased, as verified by external sources. For all analyses, two-tailed *p*-values ≤0.05 were considered statistically significant. All analyses were performed using IBM SPSS Statistics Version 26.

## Results

During the study period, a total of 911 pediatric patients who received an LT with an LLS graft were identified in the UNOS-STAR database. Of those, 508 (55.8%) underwent LT with a graft from a living donor (LD graft group) and 403 (44.2%) received a split liver graft from a deceased donor (Split graft group).

### Donor and Graft Data

Donors in the LD graft group were older when compared with donors from the split graft group (32 (IQR: 26–37) years vs. 12 (IQR: 7–17) years, respectively; *p* < 0.001). However, it is important to notice that the maximum donor age in the LD graft group was 59 years, while the older donor in the split graft group was 52 years. There was a significantly lower percentage of male donors in the LD graft group when compared to the split graft group (41.9% vs. 65.3%; *p* < 0.001). Anthropometric measurements, including weight (kg), height (cm), and BMI were significantly higher in the LD group (*p* < 0.001 for all). In congruence with the nature of the groups, CIT was significantly shorter in the LD graft group when compared with the split graft group (1.5 (1.0–2.3) hours vs. 7.5 (6.2–9.0) hours; *p* < 0.001) (Table 1).

**TABLE 1:** Donor characteristics according to donor type.

Donor variables	LD graft group (*n* = 508)	Split graft group (*n* = 403)	*p*-value
Donor age (years)	32 (26–37)	12 (7–17)	<0.001
Donor Male Gender (%)	213 (41.9)	263 (65.3)	<0.001
Donor weight (kg)	70.6 (61.3–83.0)	48.8 (25–64)	<0.001
Donor height (cm)	167.6 (162.5–175.2)	154.9 (124–170)	<0.001
Donor BMI	24.9 (22.5–28.0)	20 (16.8–22.6)	<0.001
Cold Ischemia Time (hours)	1.51 (1.0–2.33)	7.5 (6.2–9.0)	<0.001

Median (IQR). Abbreviations: BMI, body mass index; LD, living donor.

### Recipient Preoperative Data

Age at transplant and anthropometric measurements were similar between groups. Baseline characteristics such as the presence of ascites, and history of PVT were also similar. More patients in the LD group had a history of previous abdominal surgery (306 (60.2%)) vs. the split graft group (193 (47.9%)) (*p* = 0.001). PELD scores at the time of listing and LT were similar between groups. However, fewer recipients were listed under status 1 in the LD graft group compared with the split graft group (66 (13%) vs. 96 (23.8%); *p* < 0.001). The most common indication for pediatric LT was biliary atresia, occurring in 57.2% of recipients in the LD group, and in 38.9% of recipients in the split graft group. The LD group had significantly longer time spent on the waitlist (LD group = 60 (22–138) days vs. split graft group = 46 (16–108) days) (Table 2). The longer waitlist time was still observed after excluding recipients with status 1 (LD group *n* = 442, 69 (30–154) days, vs. Split graft group *n* = 307, 56 (21–132) days, *p* = 0.027).

**TABLE 2:** Recipient characteristics according to donor type.

Recipient variables	LD graft group (*n* = 508)	Split graft group (*n* = 403)	*p*-value
Age at transplant (years)	1 (0–3.7)	1 (0–3)	0.44
Male Gender (%)	246 (48.4)	205 (50.9)	0.46
Anthropometrics at transplant			
Weight (kg)	9.1 (7.0–17.17)	9.6 (7.0–15.7)	0.08
Height (cm)	73 (65.0–98.8)	74 (64–97)	0.35
BMI at transplant	16.7 (15.5–18.2)	16.8 (15.4–18.3)	0.59
Lab values at transplant			
INR	1.4 (1.1–2.0)	1.4 (1.1–2.2)	0.14
Albumin	3.1 (2.6–3.7)	3.1 (2.6–3.7)	0.79
Creatinine	0.20 (0.20–0.31)	0.27 (0.20–0.38)	0.002
Total bilirubin	9.3 (2.1–17.9)	6.6 (1–16.8)	0.005
Ascites			
Absent (%)	143 (28.1)	120 (29.8)	0.14
Slight (%)	99 (19.5)	59 (14.6)	
Moderate (%)	52 (10.2)	55 (13.6)	
N/A (%)	213 (41.9)	169 (41.9)	
PVT history (%)	15 (3.0)	15 (3.7)	0.33
Previous upper abdominal surgery (%)	306 (60.2)	193 (47.9)	0.001
Diagnosis			
Biliary atresia (%)	291 (57.2)	157 (38.9)	<0.001
Metabolic diseases (%)	44 (8.6)	76 (18.8)	
Tumor related (%)	24 (4.7)	41 (10.1)	
Acute liver failure (%)	38 (7.4)	39 (9.6)	
Cholestatic disorders (%)	19 (3.7)	25 (6.2)	
Other cirrhotic (%)	78 (15.3)	62 (15.3)	
PSC (%)	14 (2.7)	3 (0.7)	
PELD at listing	11 (2–20)	12 (0–21)	0.77
Calculated PELD at transplant	16 (5–25)	16 (4–24)	0.28
Status 1 (%)	66 (13)	96 (23.8)	<0.001
Time on wait list (days)	60 (22–138)	46 (16–108)	0.007

Median (IQR). Abbreviations: BMI, body mass index; INR, international normalized ratio; LD, living donor; N/A, not applicable; PELD, pediatric end-stage liver disease score; PVT, portal vein thrombosis; PSC, primary sclerosing cholangitis.

### Postoperative Outcomes

Length of hospital stay was significantly shorter in the LD graft group (16 (11–26) days vs. 20 (13–33) days, respectively; *p* < 0.001). The overall graft failure rate was significantly lower in the LD graft group (LD group = 9.8% vs. Split graft group = 14.6%; *p* = 0.027). The most common cause of graft failure in both groups was HAT, which occurred at similar rates between groups (Table 3). Other vascular thrombosis as the cause of graft failure were more common in the LD group (LD group = 13 (2.6%) vs. Split graft group = 7 (1.7%); *p* = 0.046). PNF rate was significantly lower in the LD group (LD group = 4 (0.8%) patients vs. Split graft group = 6 (1.5%) patients; *p* = 0.01). Diffuse cholangiopathy was the cause of graft failure in 1 patient who received a split graft and did not occur in any LD recipients (*p* = 0.036). Other reported causes of graft failure can be found in Table 3. Analysis of graft failure and important causes within 30-days, 90-days and over, yielded no significant difference in rates among the LD donor group vs. the split graft group (Table 3).

**TABLE 3:** Post-operative outcomes according to donor type.

Postoperative outcomes	LD graft group (*n* = 508)	Split graft group (*n* = 403)	*p*-value
LOS post LT (days)	16 (11–25)	20 (13–33)	<0.001
Graft failure (%)	50 (9.8)	59 (14.6)	0.027
<30-days	23 (46)	26 (44.1)	0.49
>30–90 days	3 (6)	4 (6.8)	0.59
>90-days	24 (48)	29 (49.2)	0.52
Graft failure causes			
Hepatic artery thrombosis (%)	13 (2.6)	11 (2.7)	0.19
<30-days	8 (61.5)	7 (63.6)	0.62
>30–90 days	0	0	
>90-days	5 (38.5)	4 (36.4)	0.62
Other vascular thrombosis (%)	13 (2.6)	7 (1.7)	0.046
<30-days	11 (84.6)	7 (100)	0.52
>30–90 days	0	0	
>90-days	2 (15.4)	0	0.41
Portal vein thrombosis (%)	6 (1.2)	5 (1.2)	0.74
<30-days	4 (66.7)	4 (80)	0.57
>30–90 days	0	0	
>90-days	2 (33.3)	1 (20)	0.62
Infection (%)	3 (0.6)	5 (1.2)	0.39
<30-days	1 (33.3)	1 (20)	0.64
>30–90 days	0	0	
>90-days	2 (66.7)	4 (80.0)	0.67
Biliary related (%)	1 (0.2)	2 (0.5)	0.54
<30-days	0	0	
>30–90 days	0	0	
>90-days	1	2	0.54
Diffuse cholangiopathy (%)	0	1 (0.2)	0.036
Hepatitis *de novo* (%)	0	1 (0.2)	0.31
Recurrent disease (%)	2 (0.4)	2 (0.5)	0.44
Hepatic outflow obstruction (%)	1 (0.2)	0	0.63
Primary Non-Function (%)	4 (0.8)	6 (1.5)	0.017
Re-transplant (%)	28 (5.5)	31 (7.7)	0.18
Mortality (%)	21 (4.1)	27 (6.7)	0.08
1-/3-/5-y graft survival (%)	93/89/85	90/86/79	0.058
1-/3-/5-y patient survival (%)	97/95/95	95/93/91	0.11

Median (IQR). Abbreviations: LT, liver transplant; LD, living donor; LOS, length of hospital stay.

**FIGURE 1:**
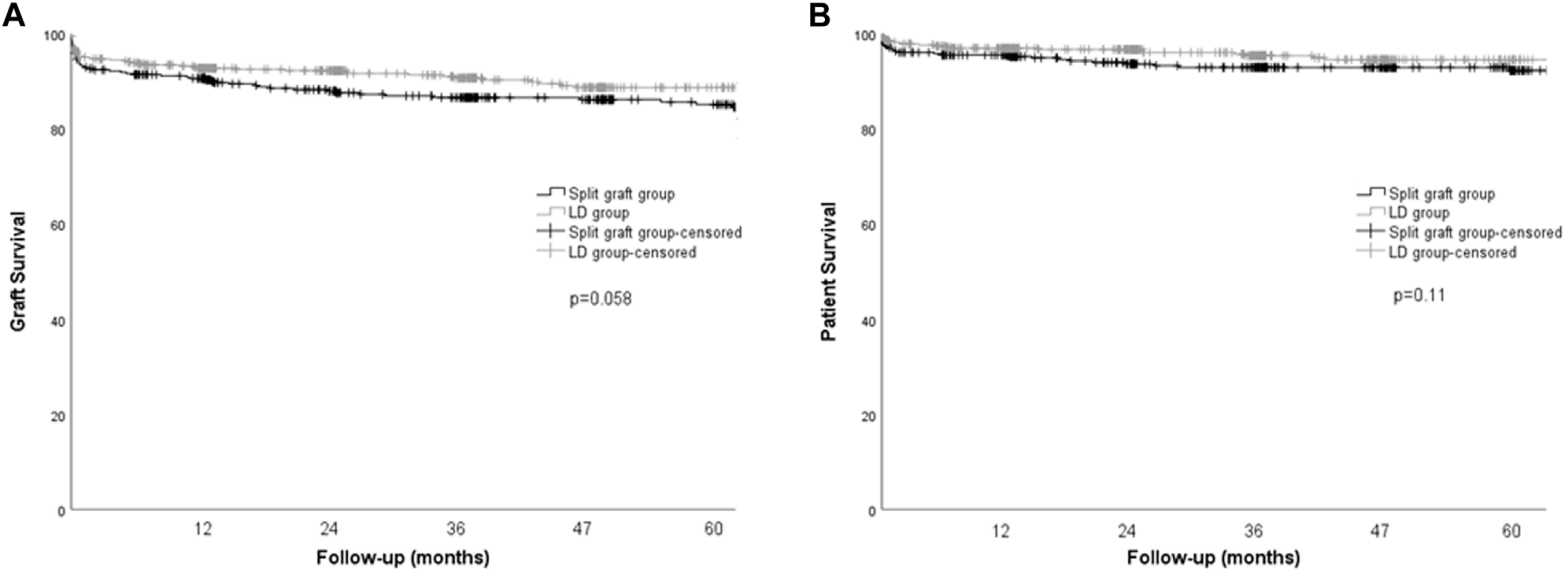
Kaplan-Meier curves for graft and patient survival in pediatric recipients according to donor type. **(A)** Graft survival, *p* = 0.058, log rank (Mantel-Cox). **(B)** Patient survival, *p* = 0.11, log rank (Mantel-Cox).

Re-transplantation rates and mortality were not significantly different between groups. Graft and patient survival at 1-, 3-, and 5-years were also similar between the LD and split graft groups (Figure 1).

### Subgroup Analysis: Recipients With Biliary Atresia

To identify significant factors influencing outcomes amongst groups, using a more homogeneous sample we performed a subgroup analysis of recipients with biliary atresia receiving a graft from a LD (*n* = 291) vs. those receiving a split liver graft (*n* = 157). Baseline recipient characteristics were similar between groups. Importantly, in this subanalysis time spent on the waitlist was similar in both groups (69 (34–152) days in the LD group vs. 67 (31–125) days in the split graft group; *p* = 0.34). The proportion of male donors was lower in the LD group vs. the split graft group (40% vs. 69%, respectively; *p* = <0.001). Additional donor characteristics appear in Table 4. As seen in the main analysis, LOS was significantly shorter in the LD group (LD group = 16 (11–24) days vs. split graft group = 20 (13–33) days, *p* = <0.001). Although improved 1-, 3- and 5-years graft survival was found in the LD group (96/94/94%, vs. 90/89/81; *p* = 0.004), patient survival at 1-, 3-, and 5-years remained comparable between groups (98/97/97% vs. 94/93/92%; *p* = 0.055).

**TABLE 4:** Subgroup analysis on pediatric recipients with biliary atresia diagnosis according to donor type.

	LD group (*n* = 291)	Split graft group (*n* = 157)	*p*-value
Donor variables			
Donor age	32 (26–37)	11 (6.5–15)	<0.001
Donor male gender (%)	115 (39.5)	109 (69.4)	<0.001
Donor weight (kg)	69.8 (61.2–83)	39.9 (23.5–60)	<0.001
Donor height (cm)	167.6 (162.5–175.2)	149 (122.5–165)	<0.001
Donor BMI	25 (22.6–27.8)	19 (15.9–21.2)	<0.001
Cold ischemia time (hours)	1.5 (1–2.4)	7.5 (6.2–9.3)	<0.001
Recipient variables			
Age at transplant	0 (0–1)	0 (0–1)	0.12
Male gender (%)	127 (43.6)	69 (43.9)	0.95
Anthropometrics at transplant			
Weight (kg)	7.7 (6.4–10.4)	7.7 (6.3–9.9)	0.68
Height (cm)	67.4 (63.4–78.5)	67.5 (62.7–75)	0.38
BMI at transplant	16.6 (15.4–18.2)	16.8 (15.4–18.2)	0.55
Lab values at transplant			
INR	1.4 (1.1–1.9)	1.4 (1.2–1.9)	0.19
Albumin	3 (2.5–3.3)	2.8 (2.4–3.3)	0.35
Creatinine	0.2 (0.1–0.3)	0.2 (0.1–0.3)	0.32
Total bilirubin	11.2 (5–18.3)	11.9 (4–18)	0.88
Ascites			0.14
Absent	57 (19.6)	31 (19.7)	
Slight	65 (22.3)	30 (19.1)	
Moderate	33 (11.3)	30 (19.1)	
N/A	136 (46.7)	66 (42.0)	
PVT history	8 (2.7)	5 (3.2)	0.75
Previous upper abdominal surgery	249 (85.6)	134 (85.4)	0.9
PELD at listing	12.5 (5.7–19)	14 (9–19)	0.62
Calculated PELD at transplant	17 (9–25)	18 (10–23)	
Status 1	8 (2.7)	9 (5.7)	0.11
Days on waitlist	69 (34–152)	67 (30–124)	0.34
Postoperative outcomes			
LOS (post-tx)	16 (11–24)	19 (14–33)	<0.001
Hepatic artery thrombosis	3 (1)	5 (3.2)	0.02
Other vascular thrombosis	3 (1.9)	4 (1.4)	0.03
Primary non-function (%)	1 (0.3)	1 (0.6)	0.004
Re-transplant	6 (2.1)	10 (6.4)	0.01
Mortality	7 (2.4)	10 (6.4)	0.03
1-/3-/5-y graft survival (%)	96/94/94	90/89/81	0.004
1-/3-/5-y patient survival (%)	98/97/97	94/93/92	0.055

Median (IQR). Abbreviations: LT, liver transplant; LD, living donor; LOS, length of hospital stay.

### Subgroup Analysis: Recipients ≤10 kg

To assess the outcomes following LT in small pediatric recipients, a subgroup analysis including patients with a body weight ≤10 kg was performed. During the study period, a total of 487 LT were performed in pediatric patients with a body weight ≤10 kg. From this, while 275 (56.5%) were performed with an LD graft, 212 (43.5%) were performed using a split LLS graft from a deceased donor. Patients in the LD group spent more time on the waitlist (LD group = 52 (23–109) days vs. Split graft group = 43 (14–95) days; *p* = 0.02). As in the main analysis, fewer patients in the LD graft group were listed as status 1 when compared to recipients in the split graft group (24 (8.7%) patients vs. 43 (20.3%) patients, respectively; *p* = <0.001).

Graft survival at 1-, 3-, and 5-years was significantly higher in the LD group (94%, 90%, 88%) vs. the split graft group (89%, 85%, 76%; *p* = 0.011). Patient survival at 1-, 3-, and 5-years was similar between groups (Table 5).

**TABLE 5:** Subgroup analyses on pediatric recipients according to donor type for smaller recipients.

	Recipients ≤10 kg			Recipients ≤10 years		
Recipient variables	LD graft group (*n* = 275)	Split graft group (*n* = 212)	*p*-value	LD graft group (*n* = 451)	Split graft group (*n* = 375)	*p*-value
Wait list time (days)	52 (23–109)	43 (14–94.7)	0.02	57 (22–131)	47 (16–107)	0.03
1-/3-/5-y graft survival (%)	94/90/88	89/85/76	0.011	94/90/88	91/86/78	0.015
1-/3-/5-y patient survival (%)	97/95/95	94/91/89	0.06	97/95/95	96/93/91	0.16

Median (IQR). Abbreviations. LD, living donor.

### Subgroup Analysis: Recipients ≤10 Years Old

Recipients ≤10 years old (LD group = 451 vs. Split graft group = 375) were also analyzed. Again, the LD graft group had significantly longer wait times (LD group = 57 (22–131) days vs. Split graft group = 47 (16–107) days; *p* = 0.03). Fewer patients in the LD graft group were status 1 at the time of transplant when compared with patients in the split graft group (59 (13.1%) vs. 86 (22.9%), respectively; *p* < 0.001). Graft survival at 1-, 3-, and 5-years was significantly higher in the LD group (94%, 90%, 88%) than the split graft group (91%, 86%, 78%) (*p* = 0.015). Patient survival at 1-, 3-, and 5-years was similar between groups (Table 5).

## Discussion

This study compares UNOS data on LDLT and SLT using the left lateral segment in the pediatric population within the last decade. Our analysis revealed improved post-operative outcomes including shorter hospital stays and lower rates of graft failure in living donor recipients. Our study also revealed that patients with a diagnosis of biliary atresia, those who weighed <10 kg or were <10 years old at the time of transplant showed an improved graft survival at 1-, 3-, and 5-years when they received an LLS graft from a living donor.

Previous studies including both graft types have shown acceptable graft and patient outcomes with living donor and split grafts compared to whole liver grafts (6,9, 12-14). A study by Mogul et al. comparing 15-years trends in pediatric LT using the SRTR database showed improvement in LDLT and SLT in both, graft and patient survivals from 2002–2009 to 2010–2015. Unfortunately, SLT and LDLT outcomes were not directly compared. In the later study period of 2010–2015, 1-year survival after LDLT was higher than of whole liver transplantation (WLT), and there was no difference between SLT and WLT (9). This improvement in graft and patient survivals using these techniques over the past decade is consistent with other studies (15). In a study by Kehar et al. from the largest pediatric LT program in Canada, 1-, 5-, and 10-years graft and patient survival rates after LDLT were significantly higher than after deceased donor LT (DDLT), with no difference in surgical or medical complications (6). The graft failure rate was also higher in DDLT recipients, in accordance with our study (6). As expected, CIT in our study was significantly longer in the split graft group. This has been shown to be a predictor of prolonged stay and is associated with reduced graft function and survival (16, 17). Analysing the impact that the splitting technique used (*in situ* vs. *ex vivo*) has on CIT and therefore on graft injury and postoperative outcomes would have been interesting. However, due to the variability of the data set, this was not able to be evaluated in our study and requires further assessment in future studies. Importantly, CIT is a variable that can be modified with improved logistics between centers performing split liver transplantation and calls for a system that would allow for protection of these otherwise ideal allografts by minimizing the obstacles that extend cold ischemic times by a seven-fold difference. Indeed, the deceased donors in this study were from younger donors with significantly lower BMIs leaving extended CIT as the primary difference to explain increased graft loss, ischemic cholangiopathy, and primary non-function rates.

The appropriate lower limit of donor age for SLT has not been defined in the literature. There have been a few studies focused on evaluating the use of split livers from pediatric donors. In a study by Cescon et al., 43 livers were split from pediatric donors less than age 15. Forty left lateral segment grafts were transplanted into pediatric recipients while 39 extended right grafts were transplanted into 11 children and 28 adults (18). Two-year patient and graft-survival were similar in recipients of grafts from donors <40 kg or >40 kg, between pediatric and adult recipients, and between recipients of ERG or LLS (18). Complications rates were also similar in recipients of donors <40 kg or >40 kg (18). In a more recent study by Gao et al., the outcomes of 16 pediatric recipients of pediatric split liver grafts were analyzed. The split liver grafts came from 8 pediatric donors less than 7 years of age (19). At a 3-months follow-up, both graft and patient survival were 100%. The only surgical complication was portal vein stenosis, reported in 1 patient (19). This study also defined criteria for optimal split liver grafts in pediatric donors which includes a graft to recipient weight ratio of 2–4% (19). Thus, although pediatric split liver donors are not commonly performed, there are institutions with experience in this technique and the selection of appropriately sized recipients for the LLS and extended right grafts. Hence, despite the low average age of split liver donors in the present study, this has not been found to be associated with higher rates of complications in the literature (18, 19).

There is an association between decreased wait time and waitlist mortality in adult recipients after LDLT (20, 21). In the pediatric community, LDLT has been supported because of its potential to decrease wait time and waitlist mortality in this vulnerable patient population as well as the ability to perform the transplant earlier in the disease course (1). In 2019, Kehar et al. showed decreased wait times in LD recipients when the primary etiology was cholestatic liver disease, including biliary atresia. However, wait time was similar between DDLT and LDLT when all diagnoses were analyzed together (6). Opposite to what was expected or reported before, an interesting finding of the present analysis is the fact that patients receiving a left lateral segment graft from an LD spent more time on the waitlist (6, 20, 21). Wait times were significantly longer also in the additional analyses of patients <10 kg or <10 years old but not in patients with biliary atresia. This can potentially be explained by several contributing factors. First, significantly more patients receiving a split graft from a deceased donor were status 1 at the time of transplant. This implies that more patients in that study group had a higher priority on the waiting list, favoring their faster access to deceased donor grafts optimal for splitting. Also, given the higher priority of status 1 patients, an offer of a potential optimal split liver graft from a deceased donor can come up before an adequate living donor work-up is completed in a timely manner. Second, some groups might opt to work-up living donors but only proceed with living donation if no deceased donor is available in a timely manner based on recipient condition or wait to proceed with LT once recipients have grown and safely achieved an adequate size. Moreover, some groups might not consider living liver donation for critically ill patients that have faster access to deceased donor organs. Over the past decade, as the number of LDLT and SLT has increased in the pediatric population, so has the number of candidates listed as status 1A or 1B, which may have affected the difference in wait time in our analysis. We chose not to exclude status 1A patients in our study. While this may have impacted our ability to detect differences in LD and split graft groups, the large number of patients keeps our study representative of the actual transplant population (22). Lastly, the number of patients with biliary atresia was significantly higher in the LD group. Therefore, we decided to perform a subgroup analysis to evaluate the outcomes between patients with biliary atresia, finding similar results as in the main analysis, except, for a similar time on the LT waitlist between both groups.

Biliary and vascular complications in pediatric recipients have been a reason of concern when using living donors and split liver grafts. Historically, partial grafts have been associated with a higher risk of vascular complications (11, 23). Ebel et al. performed a study using multicenter data from the Society of Pediatric Liver Transplantation (SPLIT) database to evaluate the predictors of HAT. In contrast to previous publications, the authors found a decreased risk of HAT in recipients of split, reduced, or living donor grafts compared to whole grafts (OR 0.59, *p* < 0.001) (24). Furthermore, a study by Alexopoulos et al. in pediatric patients ≤7 kg undergoing liver transplant for biliary atresia showed a lower incidence of vascular thrombosis in the technical variant patients than in whole liver recipients (LD (6%) and deceased donor partial liver grafts (5%) compared with whole grafts (13%); *p* < 0.002) (25). In 2020, Boillot et al. performed a retrospective study to identify prognostic factors for 1-year graft and patient survival. Vascular complications including hepatic artery and portal vein thrombosis and or stenosis had no impact on 1-year graft or patient survival (11). Our study revealed no significant difference in the incidence of HAT or PVT but did show an increased incidence of “other vascular thrombosis” as the cause of graft failure in living donor recipients. After the subanalyses, HAT was more common in recipients with biliary atresia that received a split graft, as well as recipients who were <10 kg or <10 years old when compared with LD recipients. However, the low incidence of this cause of graft failure overall makes it difficult to draw conclusions on its implication for these particular groups of patients.

LDLT has been associated with higher rates of biliary complications in the pediatric and adult populations when compared to whole grafts (7, 26). A retrospective analysis by Laurence et al., showed no difference in the rates of biliary complications in pediatric recipients of living donor (14.6%) and deceased donor (18.4%) transplantation. In terms of surgical techniques, Roux-en-Y reconstruction was associated with lower complication rates when compared with duct-to-duct reconstruction (27). In our study using the UNOS database, there was a single reported case of diffuse cholangiopathy in a patient in the split graft group and none in the living donor group. Unfortunately, detailed data about biliary complications or more specific causes of graft failure related to the biliary system were not documented in the UNOS database, which makes it difficult to draw conclusions over this complication in this manuscript.

Subgroup analyses were performed to better understand the challenges associated with low body weight (<10 kg) of pediatric recipients and younger age at the time of transplant (<10 years old). Historically, weight above 10 kg has been predictive of graft survival and associated with improved outcomes (10, 28). Smaller recipient size increases the technical complexity of the surgery and has been associated with higher rates of vascular and biliary complications (29). However, recent studies have shown no difference in clinical outcomes, allowing for earlier transplantation in these patients (8, 30). In the previously mentioned study by Alexopoulos et al., LD and DDLT recipients ≤7 kg had superior 1-, 5-, and 10-years graft survival compared with WLT (25). In our study, graft survival at 1-, 3-, and 5-years was higher in the LD group, suggesting that LDLT may provide additional benefit to pediatric patients with low body weight.

Age at the time of transplant is also an important consideration in pediatric liver transplant outcomes. Historically, infants <12 months have the highest pretransplant mortality rate (2). In 2019, the subgroups of children less than 1 year of age (29.9%) and 1–5 years old (29.9%) were the largest age groups on the waiting list (2). A study by Byun et al. in 2014 showed no difference in survival outcomes in LDLT recipients <12 months when compared with older children (31). Our analysis showed postoperative outcomes consistent with recipients <10 kg, including improved 1-,3-, and 5-years graft survival and decreased re-transplantation in LD recipients. The decreased incidence of re-transplantation in LD recipients either <10 years old or <10 kg is an interesting finding in our study. Prognostic factors and indications for pediatric re-transplantation have been elucidated, but the association with the type of graft has not been studied to our knowledge (32, 33).

Limitations of this study include the inherent challenges of registry data. Primary diagnoses for transplant in the pediatric UNOS database were felt to be under documented and some categories redundant. The individual causes of graft failure account for a small percentage of the number of deceased donors and living donor cases included. Specifically, the “other vascular thrombosis” as a category of graft failure may have overestimated the incidence of the complication. This is further compounded by the low numbers of liver transplants occurring in pediatric patients in general over the time period. As with all registry data, the onus falls on the transplant center performing the surgery to document the correct complications and reason for graft failure. In addition, lack of detailed information limited analysis of important variables that were not recorded/available in the dataset (i.e., presence of vascular anomalies, graft/recipient weight ratios, technical complications) as well as the possibility to control for additional confounders among the study groups. However, detailed analysis and subgroup analysis, as well as the scarcity of reports in literature comparing outcomes following these techniques in pediatric recipients are amongst the strengths of the present manuscript.

In conclusion, this study demonstrates that LDLT is associated with a lower rate of graft failure in pediatric patients. Patient survival at 1-, 3-, and 5-years is comparable between LDLT and SLT. In patients with a diagnosis of biliary atresia, those with a body weight <10 kg or those <10 years old, LDLT is associated with improved graft survival and decreased need for re-transplantation. The use of LLS regardless of the type of donor could represent a safe way to facilitate access to transplantation to pediatric patients with acceptable outcomes.

## Data Availability

Publicly available datasets were analyzed in this study. This data can be found here: The data that support the findings of this study are available from the United Network for Organ Sharing (UNOS) Standard Transplant Analysis and Research file (STAR). The UNOS-STAR database does not include any patient or transplant center identifiers. Restrictions apply to the availability of these data, which were used under license for this study.

## Abbreviations

DDLT, deceased donor liver transplantation; HAT, hepatic artery thrombosis; LDLT, living donor liver transplantation; LLS, left lateral segment; PVT, portal vein thrombosis; SLT, split liver transplantation; WLT, whole liver transplantation.
